# Possible over-wintering of bluetongue virus in *Culicoides* populations in the Onderstepoort area, Gauteng, South Africa

**DOI:** 10.4102/jsava.v87i1.1371

**Published:** 2016-10-31

**Authors:** Jumari Steyn, Gert J. Venter, Karien Labuschagne, Daphney Majatladi, Solomon N.B. Boikanyo, Carina Lourens, Karen Ebersohn, Estelle H. Venter

**Affiliations:** 1 Department of Veterinary Tropical Diseases, University of Pretoria, South Africa; 2 Parasites, Vectors and Vector-Borne Diseases, Agricultural Research Council-Onderstepoort Veterinary Institute, South Africa; 3 Department of Zoology and Entomology, University of Pretoria, South Africa; 4 Equine Research Centre, University of Pretoria, South Africa

## Abstract

Several studies have demonstrated the ability of certain viruses to overwinter in arthropod vectors. The over-wintering mechanism of bluetongue virus (BTV) is unknown. One hypothesis is over-wintering within adult *Culicoides* midges (Diptera; Ceratopogonidae) that survive mild winters where temperatures seldom drop below 10 °C. The reduced activity of midges and the absence of outbreaks during winter may create the impression that the virus has disappeared from an area. Light traps were used in close association with horses to collect *Culicoides* midges from July 2010 to September 2011 in the Onderstepoort area, in Gauteng Province, South Africa. More than 500 000 *Culicoides* midges were collected from 88 collections and sorted to species level, revealing 26 different *Culicoides* species. *Culicoides* midges were present throughout the 15 month study. Nine *Culicoides* species potentially capable of transmitting BTV were present during the winter months. Midges were screened for the presence of BTV ribonucleic acid (RNA) with the aid of a real-time quantitative reverse transcription polymerase chain reaction (RT-qPCR) assay. In total 91.2% of midge pools tested positive for BTV RNA. PCR results were compared with previous virus isolation results (VI) that demonstrated the presence of viruses in summer and autumn months. The results indicate that BTV-infected *Culicoides* vectors are present throughout the year in the study area. Viral RNA-positive midges were also found throughout the year with VI positive midge pools only in summer and early autumn. Midges that survive mild winter temperatures could therefore harbour BTV but with a decreased vector capacity. When the population size, biting rate and viral replication decrease, it could stop BTV transmission. Over-wintering of BTV in the Onderstepoort region could therefore result in re-emergence because of increased vector activity rather than reintroduction from outside the region.

## Introduction

In South Africa, bluetongue (BT) occurs annually during late summer and autumn in areas with high rainfall (Verwoerd & Erasmus 2004). Outbreaks of BT usually stopped abruptly after the first frosts, resulting in apparent BT-free winters during which no clinical cases are observed (Verwoerd & Erasmus 2004). The causative virus, bluetongue virus (BTV), is therefore believed to be absent from the area or dormant in either its vector or vertebrate host. Seasonal recurrences of BTV raise the question whether the virus is re-introduced into an area or re-emerges as a result of an increase in vector capacity. Apparent over-wintering (or absence of cases during winter) of BTV is documented in South Africa (Nevill 1971) as well as Europe (Maan et al. 2008; Osmani et al. 2006; Velthuis et al. 2010), although the exact mechanism is still unknown (Singer, MacLachlan & Carpenter 2001).

Once infected, *Culicoides* vectors, although relatively short-lived, are usually infectious for the rest of their life span of up to 3 months (Purse et al. 2015; Wilson, Darpel & Mellor 2008). Several arboviruses such as Ross River virus (Lindsay et al. 1993), West Nile virus (Goddard et al. 2003; Reisen et al. 2006) and Orungo virus (Cordellier et al. 1982) have demonstrated the ability to be transmitted vertically by infected arthropod females. Apparent vertical transmission of BTV in the genus *Culicoides* has been demonstrated in *Culicoides sonorensis,* the primary North American vector, after BTV nucleic acid was detected in field-collected larvae (White et al. 2005). This study, however, did not demonstrate whether BTV RNA-positive larvae would have emerged and been able to transmit infectious virus as adults and to date there is still lack of evidence for the natural vertical transmission of BTV in *Culicoides* vectors (Osborne et al. 2015).

In most parts of South Africa climatic conditions are suitable for adult *Culicoides* midges to remain active throughout the year as temperatures seldom remain at sub-zero levels for extended periods. It is therefore possible that small populations of virus-infected adult midges might survive long enough to bridge the gap between transmission seasons during mild winters. To ensure continuous transmission of BTV, vector-free periods must be of shorter duration than the maximum period of viraemia in the vertebrate population (Mellor 1994). BTV viraemia can last up to 50 and 60 days in sheep and cattle, respectively (Koumbati et al. 1999; MacLachlan et al. 1990; Sellers & Taylor 1980). Cattle have been incriminated as amplifying and maintenance host or cycling hosts in certain parts of the country where more than 95% of cattle had antibodies specific to BTV (Steyn et al. 2015). Most species of wild ruminants and camelids are also susceptible to BTV infection, although they are frequently asymptomatic and are regarded as important reservoir hosts (Maclachlan et al. 2009). Midge-free periods must therefore be longer than 60 days to effectively break the cycle in transmission. Surveys have demonstrated the presence of large numbers of *Culicoides* midges throughout the year in areas where temperatures rarely drop below 0 °C (Meiswinkel, Venter & Nevill 2004; Venter, Nevill & Van der Linde 1997). In cooler parts of the country, adult midges have been collected during nights where the minimum temperature was as low as -4.5 °C (Venter et al. 2014). Throughout such periods, the ambient temperature is probably too low for midge emergence or viral replication within the vector, which will only occur when the temperature becomes favourable (Carpenter et al. 2011; Mellor et al. 1998; Paweska, Venter & Mellor 2002; Purse et al. 2015; Wittmann 2000).

Adult *Culicoides* midges have a lifespan of 10–20 days (Mellor, Boorman & Baylis 2000), although studies have demonstrated that their lifespan may extend during mild winter months with some individuals surviving for up to 3 months at 10 °C (Lysyk & Danyk 2007; Purse et al. 2015). It is generally accepted that survival will increase, despite lower relative humidity, with a decrease in temperature (Gerry & Mullens 2000; Mullens et al. 2004). The rate of virus replication and the minimum temperature required for replication are generally consistent for different orbiviruses across different *Culicoides* vector species (Carpenter et al. 2011). In Europe members of the Obsoletus complex can be active at temperatures as low as 3.5 °C (Glukhova 1989). In the laboratory, *Culicoides obsoletus* can survive for > 90 days and 10 days at 17 °C and 4 °C, respectively (Goffredo et al. 2004). In South Africa it was shown that *Culicoides imicola* adults can survive for > 15 days and *Culicoides pycnostictus* up to 54 days at -1.5 °C (Nevill 1971). Previous studies have also demonstrated the capability of *C. imicola* and *Culicoides bolitinos* to harbour BTV for more than 20 days at 10 °C (Paweska et al. 2002; Wellby et al. 1996). *Culicoides bolitinos* seems to be adapted to cooler temperatures than *C. imicola* (Verhoef, Venter & Weldon 2014) and able to survive the relatively mild winter temperatures as adults in Gauteng Province.

It was shown that *Culicoides* midges can, despite relatively low temperatures, occur throughout the colder winter months in some parts of South Africa (Steyn et al. 2015; Venter et al. 1997, 2014). It was also shown that the detection of viable African horse sickness virus (AHSV) in field-collected midges and in *C. imicola* in particular was linked to relatively large insect collections since no virus was detected in any pool < 1000 midges (Venter et al. 2014). Even during outbreak situations of AHSV the field infection in *C. imicola* can be as low as 0.003% (Venter, Koekemoer & Paweska 2006a). In this study the objective was to determine whether BTV could overwinter in *Culicoides* populations in the Onderstepoort area, Gauteng Province, South Africa.

## Materials and methods

### Culicoides collection and processing

The *Culicoides* midges used in this study were collected by Venter et al. (2014). Midges were collected overnight at the Agricultural Research Council-Onderstepoort Veterinary Institute (ARC-OVI) (25°39’S, 28°11’E; 1219 m a.s.l.) using 220 V down-draught Onderstepoort black light traps. Trapping was conducted one night a week from July 2010 to September 2011. Frequency of collection was increased (up to 5 nights per week) during winter months (July and August 2011). Traps were operated in the vicinity of 15–20 horses. Although not present in the vicinity of the light trap, cattle were abundant in a radius of 500 m – 750 m from the collection site. Midges were recovered from 88 black light trap collections. To preserve the *Culicoides* midges for virus isolation, collections were made into phosphate buffered saline containing calcium and magnesium (PBS [+]) to which 0.5% Savlon^®^ antiseptic had been added (Walker & Boreham 1976). After retrieval in the morning, insects were washed and stored in PBS (+) at 4 °C in the dark until they were analysed. Following species identification, the midges were removed from the PBS (+) and stored in 1.5 mL vials at -80 °C until assayed for the presence of viruses.

All the midges collected in a single week were pooled (20 to > 3000 per pool) and macerated in 1000 µL Eagle’s minimum essential medium (MEM) (Highveld Biologicals [Pty] LTD) (without serum) containing a sterile glass bead. Following maceration, insect debris was collected by centrifuging at 1300 g for 1 min after which the supernatant was added to confluent baby hamster kidney (BHK-21) cells. Samples were incubated at 37 °C in a CO_2_ incubator for 10–14 days or until cytopathic effects (CPE) were observed. Samples were passaged three times on BHK-21 cells and stored at 4 °C. Samples were numbered according to collection week (1–60).

### Ribonucleic acid extraction and real-time quantitative reverse transcription polymerase chain reaction analyses

Total RNA was extracted using an MagMAX™ Express Magnetic Particle Processor (Thermo Fisher Scientific) using the MagMAX™ Total Nucleic Acid Isolation Kit (Applied Biosystems part number AM1830). Extractions were performed according to the manufacturer’s instructions with minor changes. In brief, 2000 µL detached cell cultures were centrifuged for 5 min at 8000 g to collect the pellet. Supernatants were discarded and 300 µL Millipore ultra-pure water (18.2 Ω) was added to break the cells by means of osmosis. A total of 50 µL of sample homogenate were added to the Bead Mix≈in the row A of the 96-well processing plate wells along with 65 µL of lysis solution and 65 µL isopropanol. In rows B and C, 150 µL of Wash Solution 1 was added while Wash Solution 2 was added to rows D and E. Row F was filled with 50 µL of Elution Buffer. The extraction programme was according to the manufacturer’s instruction. Briefly, lysis binding was conducted for 5 min followed by washing with each wash solution for 30 s. The RNA was then air-dried and re-suspended in Elution Buffer.

Real-time reverse transcriptase quantitative PCR, targeting segment 10, was performed as described by Steyn et al. (2015).

## Results

### Seasonal abundance of *Culicoides* at Onderstepoort

The seasonal variation in abundance and species composition was described in detail by Venter et al. (2014). More than 500 000 *Culicoides* midges belonging to at least 26 species were collected in the 88 light traps at weekly intervals between July 2010 and September 2011. The dominant species was *C. imicola*. Despite relatively low temperatures and frost, at least 17 species, including *C. imicola*, were collected throughout winter (June – August). Although the mean number of midges per night fell from > 50 000 (March) to < 100 (July and August), no midge-free periods were recorded (Venter et al. 2014).

### Real-time quantitative reverse transcription polymerase chain reaction analyses on *Culicoides*

A total of 23 midge pools collected during 2010 (July – December) and 34 midge pools collected during 2011 (January – August) were subjected to real-time quantitative reverse transcription polymerase chain reaction (RT-qPCR) (Venter et al. 2014). Pool sizes varied between 20 and > 3000 and this was dependent on the numbers collected weekly (Venter et al. 2014). Negative results (undetectable at quantitation cycle [C_q_ > 40]) were obtained from five pools, that is, two in August 2010 and one each in October 2010, February 2011 and May 2011. All other pools – 52 out of 57 (91.2%) – tested positive for BTV RNA with C_q_ values ranging from 9.8 to 36.9 with an average C_q_ value of 29.9. Virus isolation performed previously on all of the samples demonstrated CPE in only four samples (December [*n* = 1770, *C*_q_ = 9.8], January [*n* = 1514, *C*_q_ = 10.9], February [*n* = 10 728, *C*_q_ = 34.7] and March [*n* = 13 519, *C*_q_ = 11.2]), although the virus was not identified at the time (Venter et al. 2014). The real-time RT-PCR results demonstrate that BTV may possibly be responsible for CPE.

## Ethical considerations

Materials used in the experiment posed no health risk to researchers, and no vertebrate animals were harmed. The project was approved by the research committee of University of Pretoria and funded by the Tshwane Animal Health Innovation Cluster (TAHIC) – Technology Innovation Agency (TIA) (E H Venter Grant number TAHIC12-00035). The collection of midges was financially supported by the Gauteng Department of Agriculture and Rural Development (project number: OV07/23/C231).

## Discussion

This was an extension of a study done by Venter et al. (2014) on the closely related AHSV that demonstrated a relatively low virus isolation rate in *Culicoides* midges collected at the ARC-OVI (Venter et al. 2014). No AHSV was detected during the winter although continuous adult *Culicoides* activity indicated that transmission could potentially occur. The detection of AHSV was linked to high midge numbers collected and the absence of AHSV in these midges during winter months was ascribed to the relatively low numbers of midges collected (Venter et al. 2014). Similar to AHSV, the over-wintering mechanism for BTV is unknown. The present study focused on the possibility that *Culicoides* that survived throughout winter in an area can harbour BTV and represent a possible over-wintering mechanism for the virus.

In total more than 500 000 midges were collected during the 15-month survey with species-level identification revealing 27 different species. As discussed by Venter et al. (2014), the presence of both parous and freshly blood-fed females throughout the year indicated that feeding continued throughout winter. Similar to what was concluded for AHSV, this suggests that transmission of BTV throughout the winter might be possible. The decrease in midge numbers, as determined by light trap collections during winter months, can be ascribed partly to a shift in flight activity. Midges will feed earlier (late afternoon rather than after dusk) to avoid the colder night temperatures, which would influence the numbers collected as the light traps will be less efficient during the day (Meiswinkel & Elbers 2015; Scheffer et al. 2012).

Real-time RT-qPCR was used to screen the samples for the presence of BTV RNA in the *Culicoides* population. BTV RNA was detected in 91.2% of the midge pools tested compared to 40% AHSV RNA in the same samples (Venter et al. 2014). This higher detection rate of BTV could be due to BTV outcompeting AHSV within the midge, or that BTV is predominant at the ARC-OVI due to the presence of a higher number of BTV host species such as sheep and cattle. Similar results were observed during a 6-year country-wide survey where BTV and AHSV were detected with conventional virus isolation methods (embryonated chicken eggs, baby mice, cell culture) in 11.7% and 1.5% of the midge population, respectively (Nevill, Erasmus & Venter 1992a). The difference in midge pool infection with BTV and AHSV is evident, although lower than the figures for our study. This could be because of the difference in the sensitivity of conventional viral isolation (VI) methods versus real-time PCR, where the latter is much more sensitive.

All but two of the pools that tested positive for AHSV RNA (12/35) also tested positive for BTV RNA (52/57). This demonstrates co-circulation of BTV and AHSV within the midge population and could be indicative of a possible co-infection of *Culicoides* midges.

Negative midge pools were present in all four seasons (August 2010, October 2010, February 2011 and May 2011). No distinct seasonal pattern in BTV RNA negative *Culicoides* was observed. Virus or viruses isolated in three of the four midge samples were not identified at the time, as the PCR assay yielded negative results for AHSV in those samples (Venter et al. 2014). All four VI positive samples consequently tested positive for BTV RNA (C_q_ values ranging from 9.8 to 34.7). Virus isolation positives were only detected during summer and beginning of autumn (December 2010, January to March 2011) and in only one midge pool per month. This could indicate that although BTV RNA is present throughout winter, viruses capable of replicating to infectious levels are only present in summer and early autumn.

Monitoring BTV infection in *Culicoides* midges trapped at various sites in South Africa from 1979 to 1984, Nevill et al. (1992a) showed that between 14–18 different serotypes will be encountered every season, although at varying frequencies (Gerdes 2004). Usually, three to five serotypes represented more than 60% of the total number of isolates during a specific season. These dominant serotypes are replaced by others the following season, only to regain dominance 3–4 years later (Gerdes 2004). Although this phenomenon does not support the over-wintering in adult midges, it emphasised that a number of factors will play a role in the occurrence and outbreaks of BTV. The frequency of occurrence of specific serotypes will not only depend on the *Culicoides* midges but will also be influenced by the availability of susceptible mammal hosts. The number of susceptible mammal hosts will depend on previous infection and vaccine use.

Comparing real-time RT-qPCR and VI illustrates the importance of diagnostic techniques used when determining the presence or absence of viruses. Only 7.0% of the samples tested positive with cell culture isolation techniques in comparison with 91.2% that tested positive with real-time RT-qPCR. Virus isolation detects infectious virus if the viral titre is high enough and capable of showing CPE. In contrast, real-time RT-qPCR detects viral RNA even if the virus is non-infectious. Real-time RT-qPCR is therefore important to establish whether the virus is present in a specific area or population even if it is not infectious.

It is evident that *Culicoides* midges are present throughout the year even though temperatures can fall well below the normal activity range of these insects and that viral RNA is detectable in midges throughout the year. One hypothesis is that BTV overwinters in the midges, and as soon as summer approaches and temperatures rise above 15 °C, BTV starts replicating. BTV then builds up in the midge population to sufficient levels for transmission to occur during summer and clinical disease only occurs in late summer and early autumn (Mellor et al. 2000). The increase in temperature also shortens the period between blood meals, increase feeding rate and leads to larger population sizes. Although detecting nucleic acid does not necessarily imply presence of viable viruses, our findings suggest that BTV may possibly overwinter in midges in the Onderstepoort region. Further studies are required to demonstrate conclusively that this is indeed the case.

## Conclusion

*Culicoides* midges were present throughout the 15 month survey period demonstrating that the insects are able to survive mild winter temperatures recorded at the ARC-OVI, Gauteng Province. The presence of males, freshly blood-feds and parous females during the winter months also indicate that breeding and blood feeding continues throughout winter. BTV RNA was detected in most of the midge pools and viable virus was detected in summer and autumn collections. This study demonstrates that vector-free periods are not present at Onderstepoort and therefore possible overwintering of BTV within midges in this area.
